# Symptoms timeline and outcomes in amyotrophic lateral sclerosis using artificial intelligence

**DOI:** 10.1038/s41598-023-27863-2

**Published:** 2023-01-13

**Authors:** Tomás Segura, Ignacio H. Medrano, Sergio Collazo, Claudia Maté, Carlo Sguera, Carlos Del Rio-Bermudez, Hugo Casero, Ignacio Salcedo, Jorge García-García, Cristian Alcahut-Rodríguez, Savana Research Group, José Aquino, David Casadevall, David Donaire, Judith Marin-Corral, Sebastian Menke, Natalia Polo, Miren Taberna

**Affiliations:** 1 University Hospital of Albacete, Albacete, Spain; 2 Savana Research, Madrid, Spain; 3 UC3M-Santander Big Data Institute, Madrid, Spain

**Keywords:** Computational biology and bioinformatics, Neurology

## Abstract

Amyotrophic lateral sclerosis (ALS) is a fatal, neurodegenerative motor neuron disease. Although an early diagnosis is crucial to provide adequate care and improve survival, patients with ALS experience a significant diagnostic delay. This study aimed to use real-world data to describe the clinical profile and timing between symptom onset, diagnosis, and relevant outcomes in ALS. Retrospective and multicenter study in 5 representative hospitals and Primary Care services in the SESCAM Healthcare Network (Castilla-La Mancha, Spain). Using Natural Language Processing (NLP), the clinical information in electronic health records of all patients with ALS was extracted between January 2014 and December 2018. From a source population of all individuals attended in the participating hospitals, 250 ALS patients were identified (61.6% male, mean age 64.7 years). Of these, 64% had spinal and 36% bulbar ALS. For most defining symptoms, including dyspnea, dysarthria, dysphagia and fasciculations, the overall diagnostic delay from symptom onset was 11 (6–18) months. Prior to diagnosis, only 38.8% of patients had visited the neurologist. In a median post-diagnosis follow-up of 25 months, 52% underwent gastrostomy, 64% non-invasive ventilation, 16.4% tracheostomy, and 87.6% riluzole treatment; these were more commonly reported (all Ps < 0.05) and showed greater probability of occurrence (all Ps < 0.03) in bulbar ALS. Our results highlight the diagnostic delay in ALS and revealed differences in the clinical characteristics and occurrence of major disease-specific events across ALS subtypes. NLP holds great promise for its application in the wider context of rare neurological diseases.

## Introduction

Amyotrophic lateral sclerosis (ALS) is a fatal, neurodegenerative motor neuron disease of unknown etiology^1–3^. The clinical manifestations of ALS include muscle weakness, limb paralysis, and bulbar and corticobulbar symptomatology (e.g., dysphagia, dysarthria, tongue wasting) due to the progressive degeneration of upper and lower motor neurons^1,3^. In ALS, the severity of the symptoms worsens rapidly over time. Indeed, it has been estimated that about half of patients with the disease die within the first 2- or 3-years following diagnosis, usually from respiratory complications^1,4^.

Although ALS was originally described more than 150 years ago by the French neurologist Charcot^5^, our current understanding of the disease is relatively limited. Consequently, clinicians lack diagnostic tools and effective therapeutic options to halt its progression. The development of effective strategies for the management of ALS requires novel insights into the pathogenesis of the disease, the discovery of diagnostic biomarkers for early detection, and a thorough description of patients’ clinical characteristics^2^. Because the limited therapeutic options available are more effective at the initial stages of the disease, an early diagnosis is crucial for longer survival rates^6^.

With an estimated global prevalence ranging between 4.1 and 8.4 per 100,000 individuals^7^, ALS is considered a rare disease. From a clinical standpoint, diseases with low prevalence are best understood using population-based registries with available follow-up information across large numbers of patients^2,8,9^. A paramount source of real-world data (RWD) with these features is the clinical information in patients’ Electronic Health Records (EHRs). Particularly, the extraction and analysis of the unstructured clinical information in EHRs using artificial intelligence and machine learning tools (most notably Natural Language Processing, NLP) has yielded novel insights into patients’ clinical characteristics, disease management, prognosis, and epidemiological trends in different therapeutic areas^9–16^.

Using the *EHRead®* NLP technology^9,11,15–19^ to analyze the unstructured clinical information in EHRs, this study aimed to identify ALS patients from the entire source population in the SESCAM Healthcare Network (Castilla-La Mancha, Spain) to (a) characterize their demographic and clinical profile, (b) determine the delay between symptom onset and diagnosis, and (c) determine the timing of disease-specific clinical events during the course of the disease.

## Materials and methods

### Ethical standards

This study was classified as a ‘non-prospective post-authorization study’ (EPA-OD) by the Spanish Agency of Medicines and Health Products (AEMPS) and was approved by the Ethics Committee for Research with medicinal products (ECRmp) of the Integrated Healthcare System Management Office of Albacete (Protocol ID: SES-BAC-2019-01). All methods and analyses were compliant with local legal and regulatory requirements, as well as generally accepted research practices described in the Helsinki Declaration in its latest edition. Data were analyzed from de-identified EHRs, which were aggregated in an irreversible, dissociated manner. For this reason, individual patient consent for participation in the study was not required and thus waived by the ECRmp that evaluated the study.

### Study population

The study population comprised all adult patients with at least two mentions of ALS diagnosis in the EHRs within the study period (January 1, 2014, and December 31, 2018). The diagnostic criteria for ALS considered here, as followed during routine clinical practice, are aligned with El Escorial guidelines by using all previously described levels in both EEC and rEEC (*El Escorial Criteria* and *revised El Escorial Criteria, respectively*)^20,21^. Thus, patients with ‘clinically definite’, ‘clinically probable’, ‘clinically probable—laboratory-supported’, ‘clinically possible’, and ‘clinically suspected’ ALS were included in the study.

### Study design

**Figure 1:**
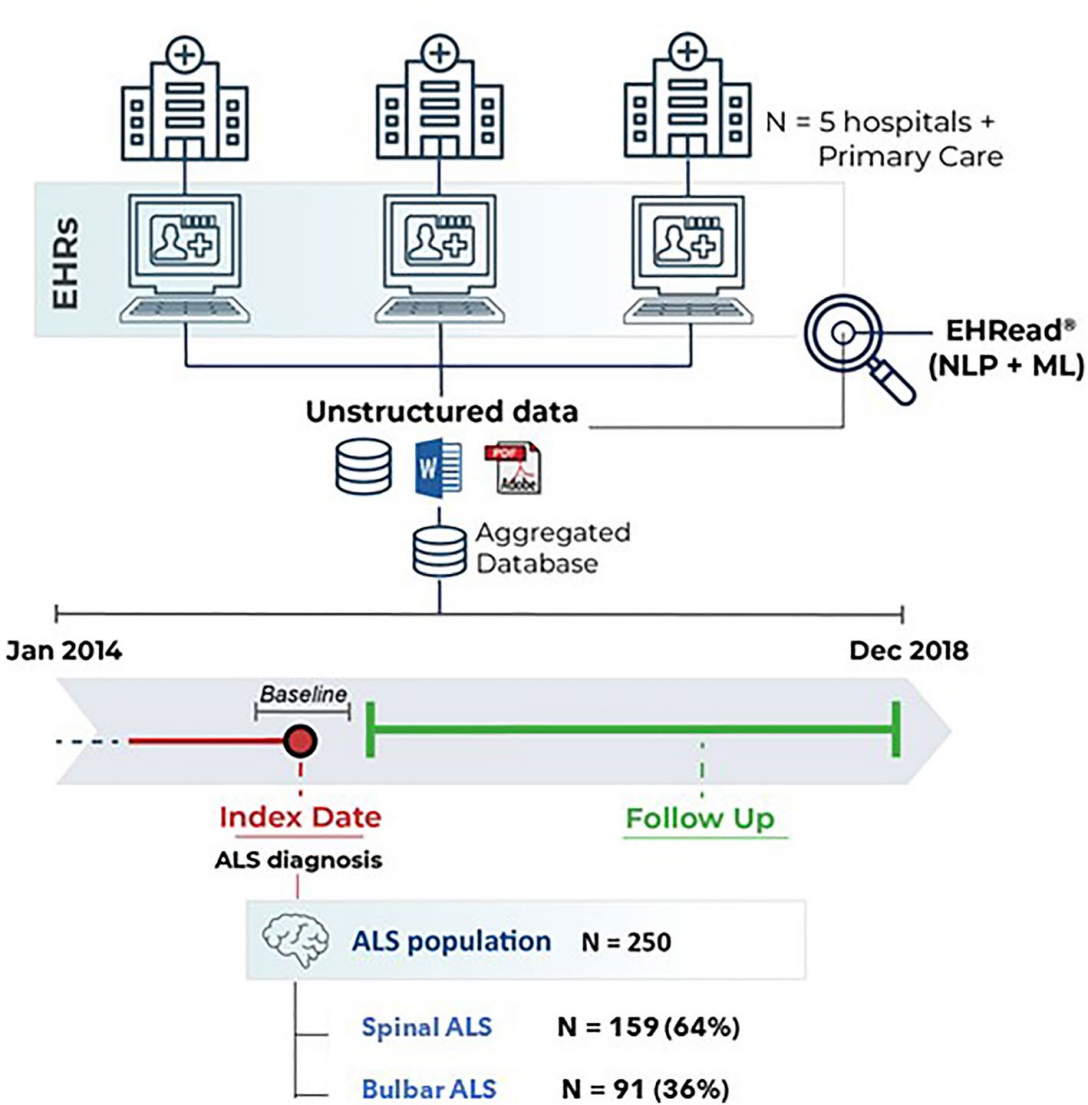
Study design and population. Using the EHRead® technology, the unstructured clinical data from patients’ EHRs were extracted and analyzed at two different time windows, namely Index Date and Follow Up (see Methods for details). From a source population of all attended individuals in the participating hospitals at least once during the study period, a total of 250 patients diagnosed with ALS were identified. *EHRs* electronic health records, *ML* machine learning, *NLP* natural language processing; *ALS* amyotrophic lateral sclerosis.

This was a retrospective and multicenter study based on the secondary use of the clinical information in the EHRs of the participating hospitals. A cross-sectional analysis of all patients was conducted at the time of inclusion in the study, hereafter referred to as index date (Fig. 1). For all patients, the index date (i.e., diagnosis date) was defined as the timepoint when the ALS diagnosis is first mentioned in the EHRs within the study period (January 1, 2014, and December 31, 2018); of note, patients diagnosed outside the study period were excluded from further analyses. The follow-up comprised the period between the index date and the last EHR available during the study period.

### Data source

The unstructured, free-text clinical information from EHRs was extracted from Primary Care services and 5 representative hospitals within the SESCAM Network (namely University General Hospitals of Toledo, Guadalajara, Albacete, Ciudad Real, and Cuenca) (Fig. 1). Structured data from Hospital Pharmacy was also included in the analyses. The source population comprised all patients attended at least once during the study period in any of the participating sites. Data were collected from all available services and departments in each participating site, including emergency, external consultations, and hospitalization notes.

### Extracting the unstructured information from EHRs

Using the *EHRead®* technology^9,11,15–19^, based on NLP and machine learning, the clinical concepts captured in patients’ EHRs were extracted and subsequently standardized into a SNOMED-CT-based terminology^22^. Once extracted from the free-text narratives in EHRs and translated into a common terminology, data were converted into a synthetic database using several steps (i.e., the NLP pipeline). A first pre-processing step involved cleaning the raw text to prepare it as a valid input for NLP models. Then, a Name Entity Recognition (NER) detection module for EHR sections and a Temporality NER module to organize the extracted information in time were applied. The pipeline also included Name Entity Disambiguation (NED) for acronyms and specific modules to detect whether statements were clinical confirmations, negations, or speculations. A relationship module linked the Temporality entities to the main NER entities. Finally, the last step comprised an internal medical verification for data completion and accuracy done by a medical team specialized in NLP with proven experience with the EHRead*®* technology.

The ability of the NLP system to properly identify EHRs containing key variables associated with ALS was externally assessed according to previously published procedures^19^ (see Supplemental Methods for details). Briefly, this external validation consisted of a comparison between the reading output of the NLP system and an annotated corpus of medical records by expert physicians in the SESCAM (i.e., the ‘gold standard’). These metrics are expressed in terms of precision, recall, and their harmonic mean F1-Score.

### Data analyses

Categorical variables are described via frequency tables; numerical variables are presented using summary tables that include the mean, standard deviation (SD), median, and interquartile range (Q1, Q3). The relative percentage of missing data and number of non-evaluable outcomes are also shown for each variable. Lack of information (i.e., unavailable data in patients’ EHRs) was considered a ‘true zero’ for binary variables (e.g., absence of a comorbidity) but was treated as missing data for numerical variables (e.g., laboratory values). The analysis of included variables was performed using three temporal windows during the course of the disease. Pre-diagnosis and baseline data were analyzed using a window between  − 36 and − 3 months, and − 3 and + 1 months around index date, respectively. These data were analyzed in patients with at least 1 year of available pre-diagnosis information. Follow-up analyses were performed considering the time span from + 1 month following diagnosis to the last available datapoint in patients’ EHRs within the study period. Results are presented separately for all patients and by ALS subtype, namely Spinal and Bulbar ALS. Distinction between sporadic and familial cases was possible, however the results were not considered because of the reduced number of familial patients. To statistically compare data across ALS subgroups we proceeded as follows. For dichotomous variables, we tested the null hypothesis that the proportions are equal using Pearson’s chi-squared tests. Yates’ continuity correction was applied when any observed absolute frequency was less than 5; for numeric variables, we tested the null hypothesis that the means are equal using t-statistic tests, assuming different variances (Welch approximation). Finally, to visualize differences in the occurrence of disease-related clinical outcomes after diagnosis (namely gastrostomy, non-invasive ventilation, tracheostomy, treatment with riluzole, and death) across ALS subtypes, survival curves were generated using Kaplan–Meier estimators; survival contrast between different groups was assessed using log-rank tests. It should be noted that death was considered when mortality was reflected as unstructured data in the EHRs. However, data considering tracheostomy as alternative mortality endpoint was also evaluated. All analyses were performed with *R* Software (v. 4.0.2).

## Results

EHRs from the attended population in the SESCAM Healthcare Network (Castilla-La Mancha, Spain) were processed from 5 hospitals and Primary Care services. The evaluation of the reading performance by the NLP system (see Methods) yielded a F1-Score of ≥ 0.8 for ‘ALS’ (0.89) and the main symptoms and clinical events analyzed (Table S1). Once the output quality of the NLP system was externally validated, we proceeded to analyze the study variables.

The study population comprised 250 patients diagnosed with ALS within the 5-year study period (61.6% male, mean age 64.7 ± 12.6 years); of these, 159 (64%) had spinal ALS, 91 (36%) bulbar ALS (Fig. 1). Across disease subtypes, the mean age at diagnosis was lower in spinal (63.5 ± 13.3 years) than bulbar (66.8 ± 11.3 years) ALS (P = 0.01). As shown in Table 1, the most common comorbidities across ALS subtypes at baseline were hypertension (44.0%; n = 110) and dyslipidemia (20.8%; n = 52). The distribution of diagnoses did not show any statistically significant difference across ALS subtypes.

**Table 1:** Demographic and clinical characteristics at baseline.

	ALS (all) N = 250	Spinal ALS N = 159	Bulbar ALS N = 91	P value*
Sex (%)				
Male	154 (61.6)	107 (67.3)	47 (51.6)	0.01*
Female	96 (38.4)	52 (32.7)	44 (48.4)	
Age at diagnosis				
Mean (SD)	64.7 (12.7)	63.5 (13.3)	66.8 (11.3)	0.03*
Median (Q1, Q3)	65 (58–74)	64 (56 – 73)	67 (59–75)	
Tobacco use (%)				
Smoker	26 (10.4)	20 (12.6)	6 (6.6)	0.13
Former smoker	37 (14.8)	25 (15.7)	12 (13.2)	0.58
No smoker	13 (5.2)	7 (4.4)	6 (6.6)	0.45
Unavailable**	174 (69.6)	107 (67.3)	67 (73.6)	–
Comorbidities				
Allergies	28 (11.2)	17 (10.7)	11 (12.1)	0.73
Anxiety disorder	22 (8.8)	14 (8.8)	8 (8.8)	0.99
Asthma	8 (3.2)	6 (3.8)	2 (2.2)	0.75
Cardiac arrhythmia	9 (3.6)	6 (3.8)	3 (3.3)	> 0.99
Cognitive impairment	9 (3.6)	8 (5.0)	1 (1.1)	0.21
COPD	16 (6.4)	13 (8.2)	3 (3.3)	0.21
Depression	18 (7.2)	11 (6.9)	7 (7.7)	0.81
Dyslipidemia	52 (20.8)	30 (18.9)	22 (24.2)	0.31
Heart failure	9 (3.6)	8 (5.0)	1 (1.1)	0.21
Hypertension	110 (44.0)	73 (45.9)	37 (40.7)	0.42
Hyperthyroidism	2 (0.8)	2 (1.3)	0 (0.0)	0.73
Hypothyroidism	14 (5.6)	10 (6.3)	4 (4.4)	0.73
T2D	25 (10.0)	19 (11.9)	6 (6.6)	0.17

Baseline analyses were performed using a window of [− 3, + 1 months) around diagnosis date.

*Statistically significant differences between spinal and bulbar ALS were calculated using Pearson’s chi-squared tests for categorical variables and t-statistic tests for numeric variables.

**Unavailable information as not reported in patients’ EHRs. COPD = Chronic obstructive pulmonary disease, T2D = Type 2 diabetes.

Pre-diagnosis information spanning at least 1 year prior to ALS diagnosis was available in 83.2% (n = 208) of patients. To better understand the patient journey at early stages of the disease, we aimed to determine the visits to different hospital services prior to diagnosis (Table 2). The most visited services and departments at this stage were primary care (88.4%; n = 221) and emergency room (38.4%; n = 96), whereas neurology was the most visited specialist service (38.8%; n = 97).

**Table 2:** Visited hospital departments and prior to ALS diagnosis.

	ALS (all) N = 250	Spinal ALS N = 159	Bulbar ALS N = 91	P value*
Main visited hospital services n (%)				
Primary care	221 (88.4)	138 (86.8)	83 (91.2)	0.29
Emergency	96 (38.4)	60 (37.7)	36 (39.6)	0.77
Neurology	97 (38.8)	64 (40.2)	33 (36.3)	0.53
General internal medicine	45 (18.0)	28 (17.6)	17 (18.7)	0.83
Cardiology	31 (12.4)	21 (13.2)	10 (10.9)	0.60
Traumatology and orthopedics	28 (11.2)	18 (11.3)	10 (10.9)	0.93
Respiratory medicine	28 (11.2)	18 (11.3)	10 (10.9)	0.93
Gastroenterology	23 (9.2)	14 (8.8)	9 (9.9)	0.77
Rehabilitation unit	21 (8.4)	16 (10.1)	5 (5.5)	0.21

Pre-diagnosis analyses were performed using a window of [− 3 years, − 3 months) around diagnosis date.

*Statistically significant differences between spinal and bulbar ALS were calculated using Pearson’s chi-squared tests for categorical variables and t-statistic tests for numeric variables.

Next, we analyzed the occurrence of symptoms before diagnosis and at baseline. As shown in Table 3, the most common symptoms until diagnosis were weakness (38.0%; n = 95), followed by dyspnea (21.6%; n = 54), dysarthria (15.6%; n = 39), dysphagia (14.0%; n = 35), and fasciculations (13.6%; n = 34). The distribution of pre-diagnosis symptoms was similar in all subtypes. Median (Q1, Q3) overall diagnostic delay (time from first any symptom to ALS diagnosis) was 11 (6, 18) months, similar in both spinal and bulbar subgroups (Table S2). Considering all documented symptoms, dyspnea presented a longer time to diagnosis with a median (Q1, Q3) time between symptom onset and ALS diagnosis of 12 (6, 18) months (Table 3). When dyspnea appeared as a first symptom in comparison with any other symptoms, there were a longer delay in neurologist referral as well as in diagnosis (Tables S2 and S3). Around diagnosis, the percentage of patients with documented dysarthria (15.6% vs. 31.2%), dysphagia (14.0% vs. 34.0%), and fasciculations (13.6% vs. 37.2%) showed a two-to-three-fold increase from pre-diagnosis stages (Table 3). At this stage, dysarthria was more prominent in patients with bulbar ALS patients, as compared with spinal ALS (23.3% vs. 45.1%, P < 0.001); the manifestation of other symptoms was similar across ALS subtypes.

**Table 3:** Occurrence of symptoms prior to diagnosis and at baseline.

	ALS (all) N = 250	Spinal ALS N = 159	Bulbar ALS N = 91	P value*
Symptoms prior to diagnosis n (%)				
Dysarthria	39 (15.6)	21 (13.2)	18 (19.8)	0.16
Time to diagnosis (months)^#^				
Mean (SD)	9.9 (7.1)	11.33 (8.1)	8.22 (5.6)	–
Median (Q1, Q3)	7 (5–13)	7 (6–14)	6 (4–13)	–
Dysphagia	35 (14.0)	20 (12.6)	15 (16.5)	0.39
Time to diagnosis (months) ^#^				
Mean (SD)	10.4 (8.3)	10.6 (9.8)	10.0 (6.3)	–
Median (Q1, Q3)	7 (5–12)	7 (5–12)	7 (6–13)	–
Dyspnea	54 (21.6)	34 (21.4)	20 (21.9)	0.91
Time to diagnosis (months) ^#^				
Mean (SD)	14.31 (9.1)	14.18 (9.5)	14.55 (8.6)	–
Median (Q1, Q3)	12 (6–18)	12 (6–18)	12 (8–18)	–
Fasciculations	34 (13.6)	21 (13.2)	13 (14.3)	0.81
Time to diagnosis (months) ^#^				
Mean (SD)	9.3 (7.3)	11.0 (8.4)	6.4 (4.0)	–
Median (Q1, Q3)	7 (3–12)	10 (4–13)	4 (3–10)	–
Weakness	95 (38.0)	62 (38.9)	33 (36.3)	0.66
Time to diagnosis (months) ^#^				
Mean (SD)	11.1 (7.8)	12.1 (8.4)	9.27 (6.2)	–
Median (Q1, Q3)	7 (6–14)	9 (6 – 16)	6 (5–13)	–
Symptoms at baseline n (%)				
Dysarthria	78 (31.2)	37 (23.3)	41 (45.0)	< 0.001*
Dysphagia	85 (34.0)	49 (30.8)	36 (39.6)	0.16
Dyspnea	80 (32.0)	52 (32.7)	28 (30.8)	0.79
Fasciculations	93 (37.2)	62 (38.9)	31 (34.1)	0.43
Weakness	141 (56.4)	86 (54.1)	55 (60.4)	0.32

Pre-diagnosis analyses were performed using a window of [-3 years, -3 months) around diagnosis date. Baseline analyses were performed using a window of [− 3, + 1 months) around diagnosis date.

*Statistically significant differences between spinal and bulbar ALS were calculated using Pearson’s chi-squared tests.

**Patients with at least one year of information available prior to ALS diagnosis.

^#^Time passed between first symptom occurrence and ALS diagnosis.

**Figure 2:**
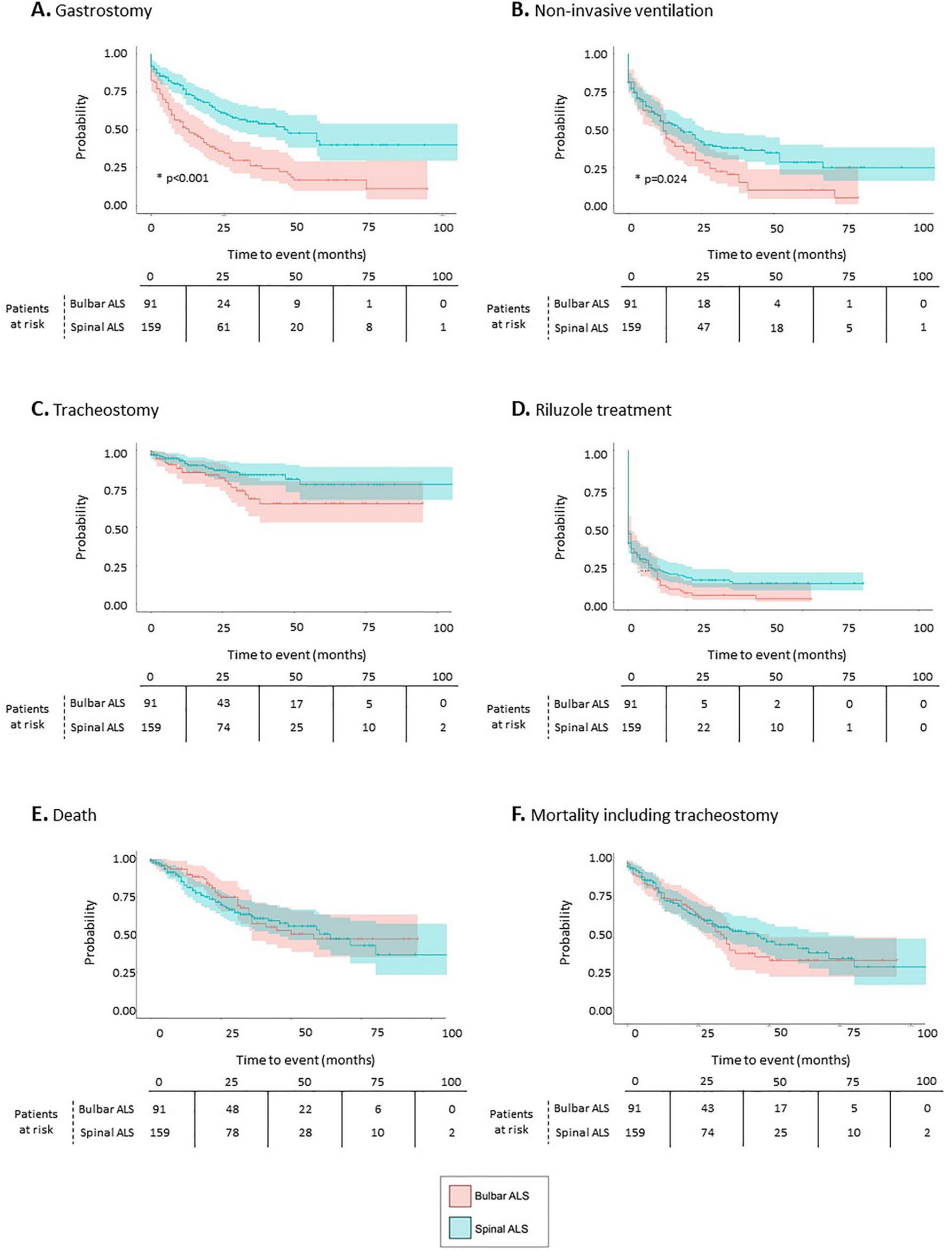
Probability of disease-specific clinical events over time during the follow-up period. Probability for gastrostomy (**A**), non-invasive ventilation (**B**), tracheostomy (**C**), riluzole treatment (**D**), death (**E**) and mortality including tracheostomy (**F**) during follow up. Data are shown for patients with bulbar ALS (orange) and patients with spinal ALS (green). The number of patients at risk (same for all categories) across the follow-up period is indicated below. Shaded areas indicate confidence intervals (CI). *Denotes significant difference across subgroups (P < 0.05).

Finally, we sought to determine the time span between ALS diagnosis and disease-specific clinical events, including procedures (gastrostomy, non-invasive ventilation, and tracheostomy), pharmacological treatment (riluzole), and mortality. As shown in Table 4, the median (Q1, Q3) duration of follow up was 25 (11, 43) months. Across ALS subtypes during follow up, 52% (n = 130) underwent gastrostomy, 64% (n = 160) non-invasive ventilation, 16.4% (n = 41) tracheostomy, and 87.6% (n = 219) treatment with riluzole. These procedures were more frequent in patients with bulbar ALS (all Ps < 0.05). Death was documented in 34.8% (n = 87) patients [44.0% (n = 110) when tracheostomy was also considered as a mortality endpoint], with a median (Q1, Q3) time since ALS diagnosis of 19 (10, 31) months. The probability for these clinical outcomes in each ALS subtype across the follow-up period is shown in Fig. 2. Significant differences in the probability of suffering an event across time in both ALS subtypes were observed for gastrostomy (Fig. 2A; P < 0.001) and non-invasive ventilation (Fig. 2B; P = 0.024), which were greater in bulbar ALS.

**Table 4:** Time from ALS diagnosis to relevant disease-specific events during follow-up.

	ALS (all) N = 250	Spinal ALS N = 159	Bulbar ALS N = 91	P value*
Available follow up (months)				
Mean (SD)	29.5 (24.0)	28.0 (24.2)	32.1 (23.8)	0.19
Median (Q1, Q3)	25 (11–43)	23 (9–42)	26 (14–45)	
Disease-specific clinical events				
Gastrostomy n (%)	130 (52.0)	63 (39.6)	67 (73.6)	< 0.001*
Time since ALS diagnosis				
Mean (SD)	13.3 (15.43)	14.2 (15.6)	12.4 (15.2)	–
Median (Q1, Q3)	7 (1–20)	11 (1–21)	7 (1–18)	–
Non-invasive ventilation n (%)	160 (64.0)	92 (57.9)	68 (74.7)	< 0.01*
Time since ALS diagnosis				
Mean (SD)	10.68 (13.8)	10.01 (14.1)	11.59 (13.5)	–
Median (Q1,Q3)	5 (0–16)	3 (0–15)	8 (1–16)	–
Tracheostomy n (%)	41 (16.4)	20 (12.6)	21 (23.1)	0.03*
Time since ALS diagnosis				
Mean (SD)	14.8 (13.9)	14.3 (15.3)	15.3 (12.9)	–
Median (Q1, Q3)	11 (3–26)	11 (2.0–20)	11 (5–27)	–
Riluzole treatment n (%)	219 (87.6)	133 (83.6)	86 (94.5)	0.01*
Time since ALS diagnosis				
Mean (SD)	2.8 (6.4)	2.4 (6.1)	3.3 (6.6)	–
Median (Q1, Q3)	0 (0–1)	0 (0–1)	0 (0–4)	–
Death n (%)	87 (34.8)	54 (34.6)	32 (35.6)	
Time since ALS diagnosis				0.89
Mean (SD)	21.2 (17.3)	21.1 (18.9)	24.0 (14.9)	–
Median (Q1, Q3)	19 (10–31)	15 (9–28)	23 (13–35)	–
Mortality including tracheostomy n (%)	110 (44.0)	66 (41.5)	44 (48.3)	
Time since ALS diagnosis				0.29
Mean (SD)	18.8 (16.6)	19.1 (18.3)	18.3 (13.9)	–
Median (Q1, Q3)	13 (6–28)	12 (6–26)	17 (6–30)	–

Follow-up analyses were performed using from diagnosis date until last datapoint available or end of study period.

*Statistically significant differences between spinal and bulbar ALS were calculated using Pearson’s chi-squared tests for categorical variables and t-statistic tests for numeric variables.

## Discussion

This study aimed to use readily available information in EHRs using NLP tools to describe the clinical profile and timing between symptom onset, diagnosis, and relevant clinical outcomes in ALS patients automatically identified from a large source population. Our results provide a characterization of these patients and point to differences in the clinical phenotype and occurrence of major disease-specific events across disease subtypes.

The demographic characteristics (61.6% male; mean age at ALS diagnosis of 64.7 years) and the distribution of patients across ALS subtypes reported here (64% spinal ALS; 36% bulbar ALS) based on the available free-text information in EHRs are consistent with previous registry-based studies using traditional research approaches^7,23–25^. Indeed, epidemiological data across Western and Eastern geographical regions show an overrepresentation of males in the ALS population^23,26–28^, with a median age at diagnosis of 54–69 years^7,24,26,27^. Hypertension (44% of patients) and dyslipidemia (21%) were the most common comorbidities at baseline. These results are of special relevance considering the existing debate regarding the protective role of hypertension and other cardiovascular disorders in the prognosis and survival of ALS^29,30^.

Patients with ALS often debut with non-specific symptoms that may mimic other neuromuscular diseases. Since misdiagnosis is common at earlier stages and the advanced progression of the disease is necessary for clinical diagnosis, it has been estimated that the mean diagnostic delay ranges from 9 to 24 months^7,26^. Here, most patients debuted with key symptoms (namely muscle weakness, dysarthria, fasciculations, and dysphagia) with an overall diagnostic delay of 11 (6, 18) months. Notably, dyspnea was fairly common and when it was present, a longer delay until visiting the specialist (neurologist) and until diagnosis were observed. We cannot exclude that this symptom, so common in other pathologies, could have gone unnoticed or could have been attributed to other causes when appearing in very early stages of the disease. In this scenario, the analysis of large amounts of RWD using NLP tools may have revealed this antecedent as an early manifestation of ALS. In a recent study aimed at describing the epidemiology and clinical characteristics of ALS patients in a northern region of Spain using EHR data, the estimated time between symptoms onset and confirmed diagnosis was around 12 months; 40% of patients debuted with symptoms less than a year from diagnosis^25^. The patient journey seems to play a role in this delay, with early referral to a neurologist linked to shorter timing to diagnosis and improve accuracy^31,32^. However, in line with our results (with only around 40% of patients visiting the neurology department in the year prior to diagnosis), ALS patients are frequently referred to other hospital departments before the neurologist^32–34^.

Defining how fast ALS progresses is crucial to lead patients to timely care, delay disease progression, and to create a framework for the assessment of treatment efficacy in clinical-trial design^35^. In the follow-up period, 87.6% of patients in our series underwent treatment with riluzole and 16.4% of patients were assigned to tracheostomy. In line with these results, riluzole treatment has been documented in 70–90% of patients^23,36,37^ and tracheostomy rates have ranged 10–20% in different studies across European countries^37–39^. Unlike riluzole and tracheostomy, however, the percentage of patients assigned to gastrostomy and non-invasive ventilation is overrepresented in our series as compared with previous reports^23,26,37,40^. These differences may be explained by some of the clinical features of patients in our study, such as the high percentage of patients showing dysphagia and the statistically significant overrepresentation of these procedures in bulbar vs. spinal ALS during follow-up.

Regardless of treatment, the reported overall median survival time in ALS from disease onset to death ranges from 20 to 48 months^4^. Here, according to the available information in EHRs, death was documented in 35% of patients (44% when tracheostomy was considered as a mortality endpoint) over a median follow-up of 25 months. The relatively low mortality rates in a median follow-up duration of 2 years found here can be accounted for either lack of data completeness in patients’ EHRs regarding this outcome or the high percentage of patients in our series assigned to procedures such as tracheostomy or non-invasive ventilation. Indeed, previous ALS studies have considered tracheostomy as an alternative endpoint for mortality since it has been linked to survival times even longer than 5 years^27^. Similarly, the use of non-invasive ventilation^41^ and riluzole^42,43^ has been linked to improved survival in ALS.

The use of NLP and machine learning to extract and analyze the unstructured information in patients’ EHRs joins previous efforts towards the application of AI tools in ALS (for a comprehensive review, see^44^). While most of these studies used structured data from patient records such as imaging, laboratory results, or *-omics* data, the exploration of the free-text narratives in EHRs has been underrepresented in the literature. In the NLP realm, a recent study aimed at extracting real-world, unstructured textual data in records of patients with ALS to determine the sociodemographic and clinical variables associated with the need for human and technical care^45^. Given that key information in EHRs is exclusively found in the unstructured information generated during routine clinical practice^46,47^, incorporating these data into existing structured databases will undoubtedly enrich current ML-based models for ALS and define the future of NLP studies in this and other neurological diseases^35,48^.

### Strengths and limitations

To the best of our knowledge, this is the first study to extract and analyze the clinical information in EHRs in a multicentric setting to determine the timing between symptoms, diagnosis, and occurrence of key disease-related outcomes in ALS. The unbiased selection of patients included in the study guarantee the representativeness of the sample. In addition, the wide availability of pre- and post-diagnosis information allowed for an accurate longitudinal assessment of the study variables.

As with all EHR-based studies, the results presented here rely on the completeness, availability, and accuracy of the information included by physicians in patients’ records during routine clinical practice^49^. Regarding the participating hospitals, it should be noted that none of the centers had a specific ALS specialty unit in the years comprising the study period; this may have compromised the identification of all patients with a diagnosis of ALS and limited the amount of follow-up information collected in the general hospital setting. This could also explain the lack of appropriate genetic studies to confirm the presence of a familial form of the disease. However, the study describes the real daily routine in several areas across different countries which in turn adds value to the results. In a similar vein, mortality data may be incomplete since death outside the healthcare system is not always documented in EHRs in a timely manner^49^.

## Conclusion

Our results point to the occurrence of key symptoms, most notably dyspnea, weakness, dysarthria, fasciculations, and dysphagia with an overall diagnostic delay of 11 months before the first mention of ALS in patients’ EHRs; only a fourth of patients had been referred to a neurologist in the year prior to ALS diagnosis and presence of dyspnea was associated with longer delay in specialist referral and diagnosis. Our analyses also revealed differences in the clinical phenotype and occurrence of major disease-specific events (namely gastrostomy, tracheotomy, non-invasive ventilation, and riluzole treatment) during follow up across ALS subtypes. The demonstrated success of clinical NLP to extract and analyze RWE in the ALS population from patient records holds great promise for its application in the wider context of rare neurological diseases, including the deep screening of patients at risk in hospital settings.

## Supplementary Information

Supplementary Information.

## Data Availability

Data cannot be shared publicly because of contractual obligations between Savana (the research company providing the NLP system used to extract and analyze the data) and the participating hospital sites that allowed access to anonymized patient information and ultimately own the data. Individual authors did not have special access privileges to the data. Further requests regarding data availability must be sent to Savana Institutional Data Access, Marisa Serrano (mserrano@savanamed.com).
