# Supplementary material for: Symptoms timeline and outcomes in amyotrophic lateral sclerosis using artificial intelligence
Source: Sci Rep. 2023 Jan 13;13:702. doi: 10.1038/s41598-023-27863-2 (PMC9839769; doi:10.1038/s41598-023-27863-2)
Supplement: Supplementary file 1 — Supplementary Information. [file 41598_2023_27863_MOESM1_ESM.docx]

**SYMPTOMS TIMELINE AND OUTCOMES IN AMYOTROPHIC LATERAL SCLEROSIS USING ARTIFICIAL INTELLIGENCE**

**SUPPLEMENTAL MATERIALS**

**Supplemental Methods**

# Evaluation of EHRead^®^’s performance

The evaluation of the performance of *EHRead^®^* involved the following phases (**Figure S1**):

- *Text collection*. In NLP systems, the amount of data necessary to capture enough linguistic events to ensure consistent and robust performance metrics is an open question. To tackle this issue, we used Savana’s SampLe Calculator for the Evaluation (SLiCE) calculator. This calculator indicates the minimum number of annotated EHRs required to obtain the expected parameters based on the prevalence in the EHRs of the main study variable (in this case, ALS). The parameters used for this calculation include a confidence level of 95% (α = 5%), interval widths of 10% (percentage points) and expected values of precision (P) and recall (R). Thus, SliCE provides a robust estimation of P and R assuring that the true value is at ±5% (pp) with a confidence level of 95%.
- *Annotation task*. The overall goal of this phase is to evaluate the system’s accuracy when identifying records that contain mentions of ALS. To build the gold standard corpus, a set of documents was first pre-annotated using Savana’s *EHRead^®^* technology; these documents included key study variables to identify the population with ALS. Then, these documents were then corrected manually via Savana’s Evaluation Tool.
- *Annotation of the gold standard*. Two designated expert physicians (hereby referred to as ‘the annotators’) at each hospital annotated the set of randomly selected records. Annotators followed the annotation guidelines written by Savana’s medical team. Then, the Inter-Annotator Agreement (IAA) was measured using the F1-Score to ensure the consistency of the guidelines and the reliability of the annotation. The IAA is a metric that indicates the extent to which the different annotators converged in their evaluation, thus providing information regarding the difficulty of the task. Finally, a third physician acted as judge, reviewing the annotations made by the two annotators and resolving any possible discrepancies. The resulting gold standard corpus served as a resource for the evaluation of the performance of Savana technology.
- *Evaluation*. The evaluation of the system is calculated in terms of the standard metrics of Precision (P), Recall (R), and their harmonic mean F1-Score
- *Precision* =
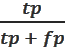
. This parameter indicates the accuracy of the system in retrieving key clinical concepts.
- *Recall* =
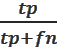
 . This parameter indicates the amount of information the system retrieves.
- *F1-Score* =
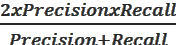
. This parameter gives us an overall performance indicator of information retrieval.

In all cases, *tp* is the number of true positives (i.e., records correctly retrieved), *fn* is the set of false negatives (i.e., records incorrectly not retrieved), and *fp* is the number of false positives (i.e., records incorrectly retrieved).

The results of *EHRead*’s performance metrics for this study are shown in **Table S1**.


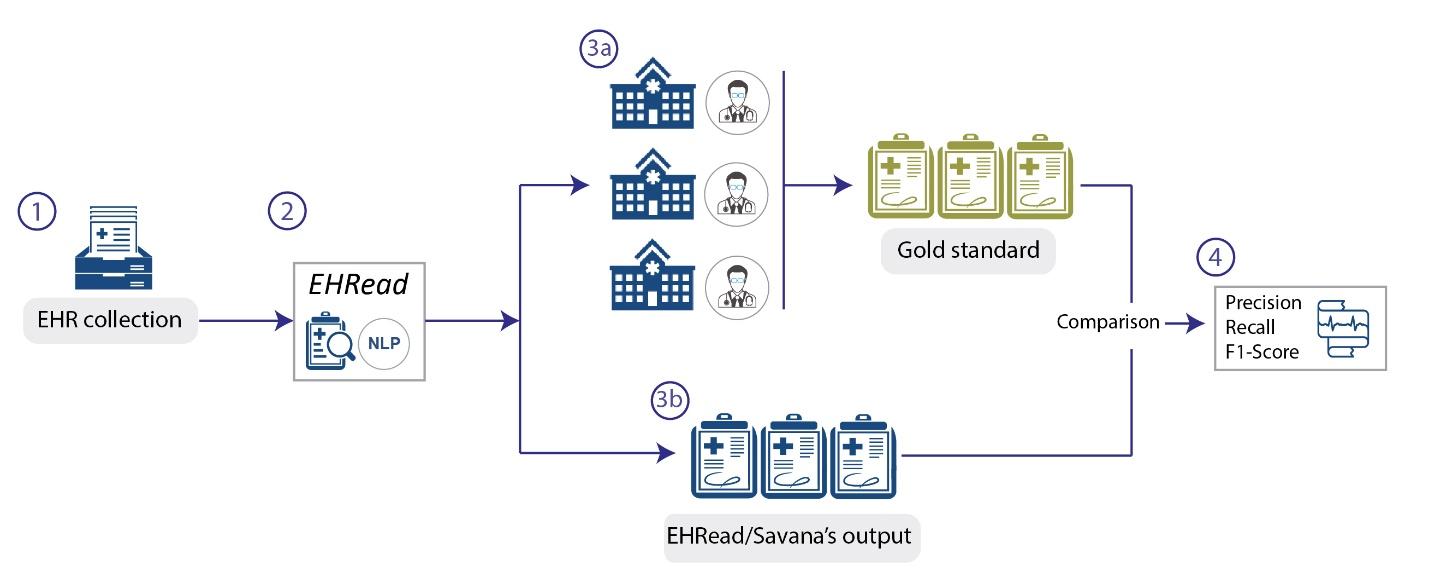


Figure S1. Assessing EHRead’s performance. Expert medical doctors annotate a subset of EHRs (1) captured via EHRead (2) to generate the ‘gold standard’ (3a). The ‘gold standard’ is then compared against EHRead’s output (3b). This comparison is expressed in terms of precision (P), recall (R), and their harmonic mean F1-Score (F1) (4). See text for further details.

**Supplemental Results**

**1. Supplemental Tables**

Table S1. Performance of EHRead^®^ identifying records that contain key study variables.

| **Variable** | **Recall (R)** | **Precision (P)** | **F1-Score** |
| --- | --- | --- | --- |
| Alzheimer’s disease | 1.00 | 0.50 | 0.67 |
| Amyotrophic Lateral Sclerosis (ALS) | 0.89 | 0.89 | 0.89 |
| Bulbar ALS | 0.75 | 0.86 | 0.80 |
| Cognitive impairment | 0.36 | 0.19 | 0.25 |
| Dysarthria | 1.00 | 1.00 | 1.00 |
| Dysphagia | 1.00 | 0.83 | 0.91 |
| Exitus (death) | 1.00 | 0.53 | 0.69 |
| Fasciculations | 1.00 | 1.00 | 1.00 |
| Gastrostomy | 0.96 | 0.82 | 0.88 |
| Invasive ventilation | 0.90 | 0.92 | 0.91 |
| Non-invasive ventilation | 0.96 | 0.96 | 0.96 |
| Respiratory failure | 0.88 | 0.88 | 0.88 |
| Riluzole | 1.00 | 1.00 | 1.00 |
| Spinal or medullar ALS | 1.00 | 0.33 | 0.50 |
| Tracheostomy | 1.00 | 1.00 | 1.00 |
| Weakness (muscle weakness) | 0.91 | 0.58 | 0.71 |
| . | | | |

Table S2. Time from first symptoms occurrence to neurologist referral and diagnosis (months)

|  | ALS (all)  n=250 | Spinal ALS  n=159 | Bulbar ALS  n=91 |
| --- | --- | --- | --- |
| Time from any symptom to Neurology visit | | | |
| N (%) | 82 (32.8) | 53 (33.3) | 29 (31.8) |
| Mean (SD) | 12.9 (8.5) | 12.7 (8.1) | 13.3 (9.2) |
| Median (Q1 - Q3) | 12 (6 - 19) | 12 (7 - 17) | 11 (5 - 20) |
| Time from any symptom to ALS diagnosis | | | |
| N (%) | 124 (49.6) | 82 (51.6) | 42 (46.1) |
| Mean (SD) | 13.1 (8.7) | 13.5 (9.0) | 12.4 (7.9) |
| Median (Q1 - Q3) | 11 (6 - 18) | 12 (7 - 18) | 11 (5 - 18) |
| Analyses were performed using a window of [-3 years, -3 months) around diagnosis date | | | |

Table S3. Time from first symptom to neurologist referral (months)

|  | ALS (all)  n=250 | Spinal ALS  n=159 | Bulbar ALS  n=91 |
| --- | --- | --- | --- |
| Dyspnea as a first symptom | | | |
| N (%) | 24 (9.8) | 17 (10.9) | 7 (7.7) |
| Mean (SD) | 12.8 (7.4) | 11.3 (5.2) | 16.3 (10.7) |
| Median (Q1 - Q3) | 12 (8 - 17) | 12 (10 - 13) | 19 (6 - 24) |
| Other symptoms as a first symptom | | | |
| N (%) | 39 (15.8) | 26 (16.8) | 13 (14.3) |
| Mean (SD) | 11.21 (8.6) | 12.04 (8.7) | 9.54 (8.3) |
| Median (Q1 - Q3) | 10 (3 - 15) | 11 (4 - 17) | 6 (3 - 12) |
| Analyses were performed using a window of [-3 years, -3 months) around diagnosis date | | | |

Table S4. Time from first symptom to ALS diagnosis (months)

|  | ALS (all)  n=250 | Spinal ALS  n=159 | Bulbar ALS  n=91 |
| --- | --- | --- | --- |
| Dyspnea as the first symptom to appear | | | |
| N (%) | 30 (12.2) | 19 (12.3) | 11 (12.1) |
| Mean (SD) | 13.4 (8.4) | 11.8 (6.7) | 16.18 (10.5) |
| Median (Q1 - Q3) | 11 (6 - 18) | 12 (7 - 15) | 11 (8 - 24) |
| Any other symptom as first symptom to appear | | | |
| N (%) | 65 (26.4) | 44 (28.4) | 21 (23.1) |
| Mean (SD) | 11.77 (8.2) | 12.7 (8.9) | 9.81 (6.2) |
| Median (Q1 - Q3) | 7 (6 - 17) | 8 (6 - 18) | 7 (5 - 13) |
| Analyses were performed using a window of [-3 years, -3 months) around diagnosis date | | | |
